# Is It Necessary to Prepare the Enamel before Dental Bleaching?

**DOI:** 10.1155/2017/5063521

**Published:** 2017-02-09

**Authors:** Andréa Dias Neves Lago, Patrícia Moreira de Freitas, Erika Michele dos Santos Araújo, Adriana Bona Matos, Narciso Garone-Netto

**Affiliations:** ^1^Department of Dentistry I, Federal University of Maranhão (UFMA), São Luís, MA, Brazil; ^2^Special Laboratory of Lasers in Dentistry (LELO), Department of Operative Dentistry, School of Dentistry, University of São Paulo (USP), São Paulo, SP, Brazil; ^3^Department of Operative Dentistry, School of Dentistry, University of São Paulo (USP), São Paulo, SP, Brazil

## Abstract

The aim of this in vitro study was to assess the influence of distinct surface treatments on the microhardness and color of enamel that will be bleached. Surface treatments are tested, accordingly: G1, no treatment; G2, 2% sodium fluoride; G3, casein phosphopeptide paste; G4, 2% fluoride+Nd:YAG laser. Forty blocks from bovine teeth composed the sample that were tested in Knoop microhardness (*n* = 10) and in color change (*n* = 10). After 24 h, bleaching with 35% hydrogen peroxide was performed for 45 min. Microhardness and color changes (using parameters Δ*E*, Δ*L*, Δ*a*, and Δ*b*) were assessed before and after bleaching. The data were analyzed by two-way ANOVA and Tukey's test (*p* < 0.05). Despite all surface treatments, a reduction of enamel microhardness occurred immediately after bleaching in all groups, being greater in G1. Enamel color changed in all groups. Immediately after bleaching, there was a decrease on enamel microhardness. However, after 7 days, some of those specimens previously treated before bleaching significantly recovered their initial microhardness without influencing the esthetic results of bleaching.

## 1. Introduction

Effects of bleaching agents on the enamel surface have been a clinical concern as they can lead to dentinal sensitivity [1], enamel demineralization [2, 3], and surface morphological changes [3–6].

Even tough professionals explain side effects of bleaching, patients still claim for this procedure. Thus, strategies which could decrease the occurrence of some of these side effects need to be further explored. Some strategies have been proposed to prevent changes in the enamel surface, such as topical fluoride application [6–8], phosphoprotein casein and calcium phosphate (CPP-ACP) [9–12], and, more recently, the use of high power lasers, such as the Nd:YAG laser [12–16]. But a question remains to be answered: will these strategies affect the effectiveness of the bleaching procedure itself?

Although the mechanism of action of fluoride in preventing caries is well known in the literature, its effect on preventing demineralization caused by bleaching agents is still controversial [6]. Fluoride, with its high affinity for calcium phosphate, contributes to the formation of complexes that precipitate on the tooth, preventing demineralization. Either way, high concentrations of fluoride in the form of varnishes and gels have shown to be effective in preventing enamel demineralization [17, 18].

Fluoride in saliva is also important. Some products have been developed to provide calcium [19–21] and phosphate, in addition to fluoride, to favor the remineralization process [20–22]. In this context, the use of casein, the predominant protein in milk serum, has been indicated to increase the availability of calcium, phosphate, and fluoride in the oral environment. The main function of phosphopeptides (CPP) from enzymatic breakdown of the casein molecules is to aggregate and stabilize ACP, amorphous calcium phosphate [20]. In addition to storing the ions that participate in the remineralization process, these molecules can adhere to the salivary film, biofilm, and oral tissues, so that the CPP-ACP complexes remain in the oral environment for a longer period [9]. When there is drop in pH, these ions are slowly and gradually solubilized and will contribute to the mechanism of remineralization [20, 23, 24].

New technologies have been described to reduce the process of demineralization when enamel is subjected to acid challenges. Since Sognnaes and Stern (1965) [25] reported that laser light increases enamel resistance to acids, many studies have been conducted to determine conditions and parameters that effectively prevent enamel demineralization [26, 27], especially with laser emission in the near-infrared range with a wavelength of 1064 nm [13, 15, 16, 27]. Recently, a clinical study reported positive results with the use of Nd:YAG laser associated with fluoride in the prevention of mineral loss from enamel [15].

Given the importance of finding new methods to prevent enamel demineralization from constant acid attacks, it is important to provide patients with safer and more conservative preventive/restorative procedures that have higher clinical durability. However, it is still not known whether these prevention alternatives affect the outcome of bleaching. Furthermore, it is not known whether these treatments can reverse possible changes in enamel microhardness without compromising the effectiveness of whitening treatment and interfering negatively in the final color of the teeth.

The objective of this in vitro study was to evaluate if previous surface treatments with fluoride, CPP-ACP, or Nd:YAG laser associated with fluoride have an influence on the microhardness of enamel, without interfering with the effectiveness of the bleaching procedure.

## 2. Material and Methods 

### 2.1. Experimental Design

This study tested two main factors: surface treatments (control, fluoride, CPP-ACP, and Nd:YAG laser associated with fluoride) and time intervals (at baseline, after surface treatment, immediately after bleaching, and 7 days after bleaching). The experimental units consisted of 60 bovine incisors that were divided into 80 dental blocks, randomly divided as follows: 40 dental blocks for Knoop surface microhardness test (*n* = 10/group) and 40 enamel blocks for analysis of color change (*n* = 10/group).

### 2.2. Preparation of Bovine Dental Blocks

Sixty bovine teeth were observed under binocular stereoscopic microscope (Olympus, Hongo, Tokyo, Japan) at 25x magnification, and those showing cracks or wear on the buccal surface were discarded. Teeth were cleaned with periodontal curettes (Duflex, SS White, Rio de Janeiro, RJ, Brazil) to remove organic debris and were submitted to cleaning procedure with pumice and water (SS White) [28]. The crowns were separated from the roots using a diamond disc (15LC series, Buehler, Lake Bluff, IL, USA) coupled to a cutting machine (Labcut 1010, Extec, Enfield, CT, USA) under water-cooling.

### 2.3. Preparation of Dental Blocks for Microhardness Evaluation

Twenty bovine teeth were cut with a double-faced diamond disc (KG Sorensen, Barueri, SP, Brazil) under water-cooling, to obtain 40 dental blocks (5 × 5 mm, from the buccal surface of the teeth) and they were used for microhardness test. Blocks were embedded in acrylic resin (Jet-Classic, Cremer, Blumenau, SC, Brazil), leaving the buccal surface exposed. After the complete acrylic resin polymerization (approximately 1 h), the blocks were planed and polished with silicon carbide sandpaper (Carbinet, Buehler, Lake Bluff, IL, USA) with decreasing grain (#400, #600, #1200, and #4000) for 30 seconds in each sandpaper [13, 29] to standardize the amount of enamel to be removed.

Finally, 1 *μ*m grain diamond paste (Buehler, Lake Bluff, IL, USA) applied with a felt disc (Texmet 1500, Buehler, Lake Bluff, IL, USA) was used for another 30 sec.

### 2.4. Preparation of Dental Blocks for Color Evaluation

Forty bovine crowns were cut with a double-faced diamond disc, under water-cooling to obtain 40 enamel blocks (10 × 10 mm), 2 mm thick. Each block was planned with an individual abrasive silicon carbide paper (#120 grit). Next, they were individually polished with silicon carbide sandpaper (Carbinet, Buehler, Lake Bluff, IL, USA) with decreasing grain (#400, #600, #1200, and #4000) for 30 seconds in each sandpaper [13, 29] to standardize the amount of enamel to be removed. Polishing was standardized using a metallic device that pressed the sample against the sandpaper.

After the end of the preparation, all samples were stored in distilled water until the beginning of the experiment.

### 2.5. Enamel Surface Treatment

The specimens of Group G1 were immersed in artificial saliva (pH 6.8) for 24 h during the period in which the treatment of the other samples (experimental groups) was carried out. The artificial saliva used consisted of CaCl_2_ 2H_2_O (0.213 g), KH_2_PO_4_ (0.738 g), KCl (1.114 g), NaCl (0.381 g), TRIS buffer (12 g), and mucin (1.2 g) (corresponding values for each 1 l of solution) [6].

Fluoride treatment (G2): a volume of 0.1 mL of 2% sodium fluoride (DFL, Rio de Janeiro, RJ, Brazil), measured in a disposable insulin syringe was applied on the buccal surface of the enamel for 1 minute. Afterwards, all the gel was carefully removed with gauze and the samples were stored in artificial saliva for 24 hours.

Treatment with CPP-ACP (G3): a volume of 0.1 mL of standard paste containing CPP-ACP (MI Paste, GC America, Chicago, IL, USA) was dispensed on the buccal surface enamel using an insulin syringe. The paste was applied on the surface for 3 minutes in a passive mode [30]. After this, all the paste was carefully removed with gauze and the samples were stored in artificial saliva for 24 hours.

Nd:YAG laser associated with fluoride (G4): fluoride gel was applied on the buccal surface of the enamel samples, as previously described in G2. Without removing the fluoride gel, samples were irradiated with laser Nd:YAG (*λ* = 1064 nm) (Lares Research, Chico, CA, USA) using the following parameters: 50 mJ of energy, power of 0.50 W at 10 Hz repetition rate, 2.5 J/cm^2^ energy density, and scanning the entire enamel surface for 30 seconds per sample [13, 15]. An optical fiber of 400 *μ*m in diameter was used, positioned perpendicularly to the enamel surface at approximately 1 mm (defocused mode). After irradiation, the samples were stored in artificial saliva for 24 hours.

### 2.6. Bleaching Technique

Twenty-four hours after completion of enamel surface treatments, samples of all groups were subjected to bleaching with 35% hydrogen peroxide (Whiteness HP, FGM, Joinville, SC, Brazil). The bleaching product contains the 35% hydrogen peroxide as active agent, thickener, red dye, and water glycol. The product was applied on the buccal surface of the enamel samples, in accordance with the manufacturer's instructions, consisting of three applications of a volume of approximately 0.1 mL for 15 minutes each, without the use of a light source to activate the whitening gel [28, 29]. After bleaching procedures, the samples were thoroughly washed under running water and dried for analyses of surface microhardness and spectrophotometry.

### 2.7. Microhardness Test

Samples were submitted to Knoop surface microhardness tests with static load of 100 g for 15 seconds (Microhardness Tester HMV 200, Shimadzu, Kyoto, Japan). Five indentations were made on the sample surface at 100 *μ*m from each other, at predetermined time intervals: M1 (baseline, control), M2 (after surface treatment), M3 (immediately after bleaching), and M4 (7 days after bleaching). The mean value of the indentations at each time interval was considered for statistical analysis. The samples were stored in artificial saliva between the different times of measurement.

### 2.8. Color Change Evaluation

The system used to measure color in the current study was developed by the CIE (Commission Internationale de L'Eclairage, 1976) [31]. Color was evaluated at four different time intervals: baseline (M1, control), after surface treatments (M2), immediately after bleaching (M3), and 7 days after bleaching (M4).

Between readouts, the samples were kept in artificial saliva to prevent dehydration and the color difference was calculated using the values of *L*^*∗*^*a*^*∗*^*b*^*∗*^ obtained after the initial readout (M1) and after each further readout (M2, M3, and M4). The color coordinates in three-dimensional system are *L*^*∗*^ = luminosity  coordinate, which is achromatic and ranges from 0 (black) to 100 (white) and the *a*^*∗*^ = chromatic coordinate green-red (−*a*^*∗*^ = green, +*a*^*∗*^ = red) and *b*^*∗*^ = blue-yellow color coordinate (−*b*^*∗*^ = blue, +*b*^*∗*^ = yellow). The total change in color (or the change in color perception) of each sample at different time intervals (Δ*E*) was calculated using the formula: Δ*E* = [(Δ*L*)^2^ + (Δ*a*)^2^ + (Δ*b*)^2^]^0.5^ for the different readout times [32].

The UV-Visible spectrometer (Cintra 10, Hitachi Model U-3501, Tokyo, Japan) requires standardized 10 × 10 mm samples to automatically read the specimens to guarantee that the readings will be performed in the same location.

In the intervals between readings, the samples were kept in artificial saliva to avoid dehydration. They were dried with absorbent paper just before the reading procedure.

### 2.9. Statistical Analysis

For both microhardness and color change evaluation, data were submitted to statistical analysis using ANOVA two-way (treatment and time intervals) followed by Tukey's test to examine statistically significant differences between groups. Minitab 17 Statistical Software was used for the statistical analysis with significance level of 5%.

## 3. Results

### 3.1. Microhardness Evaluation

ANOVA indicated statistically significant result for the factor time intervals (*p* = 0.02), indicating that, immediately after bleaching, all groups presented lower microhardness. After 7 days in saliva, samples presented an intermediate microhardness performance (Table 1). On the other hand, the main factor treatments (*p* = 0.09) and the interaction between factors (*p* = 0.99) were not statistically different (Table 2). This result indicates that previous treatments were not able to prevent the reduction in microhardness caused by bleaching, but, after 7 days of saliva storage, the microhardness was recovered.

### 3.2. Color Evaluation

For the evaluation of color change, this study has two dependent variables:

- Change of luminosity, Δ*L*, ranging from 0 to 100, featuring the chromatic spindle drives black (0) and white (100)
- Δ*E*, total color difference, within the CIE *L*^*∗*^*a*^*∗*^*b*^*∗*^, 1976, comprising the total variations of Δ*L*  Δ*b*  Δ*a*

Change of luminosity values is shown in Table 3, while the total color change is in Table 4.

There was no statistically significant difference in luminosity change regardless of the type of treatment performed in all time intervals. Considering the factor time intervals in each treatment, in G1, G2, and G4, the luminosity increased after bleaching and was maintained after 7 days of storage, but G3 showed a different performance. When, in CPP-ACP (Δ*LP*), a significant decrease in the luminosity was observed, but it did not impair bleaching (Δ*LI*) and its recovery after 7 days of storage (Table 3).

Δ*E*_*P*_ corresponded to total variation of color after treatments, Δ*E*_*i*_ immediately after the bleaching, and Δ*E*_7_ seven days after the end of the bleaching. Table 4 indicates that no statistically significant difference was observed for treatments in each time interval. Also, despite the treatment being performed or not, the bleaching procedure was effective in increasing the color change that remained changed after 7 days.

## 4. Discussion

Many studies have reported a decrease in microhardness [7, 8, 32], in addition to increased enamel porosity [33], permeability [6], and roughness [3, 11] after bleaching procedures. Therefore, the aim of this study was to find resources to minimize or even prevent these changes, such as the use of fluoride, CPP-ACP, and Nd:YAG laser associated with fluoride, when applied 24 h before bleaching.

Samples from the control group were the ones to show a reduction on microhardness at the time immediately after bleaching. These findings agree with the literature [9, 32]. Also, considering the treatments solely, it was verified that, in all experimental groups, the enamel microhardness was recovered after 7 days. This can be justified by the ability of fluoride, calcium, and phosphate ions to form a compound that is more resistant to acid attacks, since there is synergism between these ions with an artificial saliva [20–22].

Using laser in combination with fluoride increases the resistance of enamel to acids, thereby promoting a preventive effect [15, 16], because the heat promotes the dissolution of the enamel prisms and their recrystallization, thus decreasing permeability. The presence of fluoride during irradiation promotes the remineralization of enamel [14, 16].

Regarding the CPP-ACP treated samples, it is suggested that this treatment could have led to chemical changes in the tooth substrate. This could have had a negative influence on enamel brightness, because the results of this group showed decrease in brightness, while microhardness immediately after bleaching was similar to that observed after 7 days. Nevertheless, chemical tests would be required to confirm the hypothesis that CPP-ACP-induced chemical alterations in the enamel. Also, the follow-up time considered in the present study (7 days) may not be enough to make conclusions on the real effect of CPP-ACP on enamel brightness. Long-term evaluation would be necessary to evaluate it, since the subproducts of the CPP-ACP on the enamel surface could be no more existent after a higher exposure time to saliva.

It is believed that as time passes the cumulative effects of CPP-ACP with the saliva promotes adequate protection before the whitening, but 7 days before was the time we considered ideal for performing preventive measures before the dental bleaching.

Irradiation with Nd:YAG laser promotes the formation of wider enamel prisms and reduces enamel permeability [13, 14], so it is questionable whether whitening would be possible under these conditions, since the free radicals formed during the bleaching process must penetrate the tooth structure to react with the pigments. However, the results of this research did not reveal significant color alterations (Δ*E*) in the irradiated samples maybe because the bleaching agent may have been prevented from penetrating the enamel properly.

Regarding total color difference (Δ*E*), the analysis between groups showed no significant differences at different readout times. However, there was a significant color difference between all groups immediately after bleaching, which remained after 7 days. Johnston (2009) [34] reported that when Δ*E* is greater than or equal to 1, the human eye detects color differences. In this study, the Δ*E* values of all groups were not statistically different after 7 days and the color remained constant in all groups. Thus, it is possible to suggest that enamel treatments did not negatively affect the effectiveness of whitening.

Finally, further studies should be conducted, considering longer periods of use of products for surface treatment or even different products. Although it was shown that the side effects were minimized by some of the surface treatments and that they did not have any influence on the whitening potential of bleaching agents, performing these treatments 24 hours prior to enamel exposure to 35% hydrogen peroxide was insufficient to prevent the mineral changes caused by bleaching.

## 5. Conclusions

None of the treatments tested could influence enamel microhardness. But it is important to highlight that bleaching decreases microhardness, but not in a permanent way. After 7 days of saliva storage, this reduction is recovered. Also, the treatments performed did not impair bleaching effectiveness. Based on our results, we can conclude that there is no reason to perform previous treatments before bleaching to prevent the decrease in microhardness. But, if these treatments are necessary for some other reason, the substances can be applied without compromising the esthetic result of the bleaching procedure.

## Figures and Tables

**Table 1:** Mean surface Knoop microhardness (KHN) values and standard error for the factor time intervals.

Groups	Microhardness
Baseline, control (M1)	273.50 ± 6.65 A
After treatment (M2)	273.12 ± 6.65 A
Immediately after bleaching (M3)	255.77 ± 6.65 B
7 days after bleaching (M4)	266.07 ± 6.65 AB

Different letters indicate statistically significant difference.

**Table 2:** Mean surface Knoop microhardness (KHN) values and standard error for the factor treatment.

Groups	Microhardness
Control (G1)	276.47 ± 6.65 A
Fluoride (G2)	266.45 ± 6.65 A
CPP-ACP (G3)	265.67 ± 6.65 A
Fluoride + Nd:YAG laser (G4)	259.87 ± 6.65 A

Different letters indicate statistically significant difference.

**Table 3:** Means and standard deviations for the Δ*L* values.

Groups	Δ*L*_*P*_	Δ*L*_*I*_	Δ*L*_7_
Control (G1)	0.03 ± 0.46^Ab^	2.00 ± 1.55^Aa^	1.68 ± 1.32^Aa^
Fluoride (G2)	0.46 ± 1.05^Ab^	2.74 ± 1.03^Aa^	2.11 ± 1.06^Aa^
CPP-ACP (G3)	−0.26 ± 0.99^Ac^	2.71 ± 1.64^Aa^	1.83 ± 1.33^Ab^
Nd:YAG laser + Fluoride (G4)	−0.08 ± 1.23^Ab^	1.51 ± 0.35^Aa^	0.91 ± 0.58^Aa^

Different uppercase letters indicate statistically significant differences within columns.

Different lowercase letters indicate statistically significant differences within rows.

Δ*L*_*P*_: change of luminosity after treatment of enamel; Δ*L*_*I*_: change of luminosity immediately after bleaching; Δ*L*_7_: change of luminosity 7 days after bleaching.

**Table 4:** Means and standard deviations for the Δ*E* values.

Groups	Δ*E*_*P*_	Δ*E*_*I*_	Δ*E*_7_
Control	0.37 ± 0.40^Ab^	3.17 ± 2.10^Aa^	3.26 ± 1.83^Aa^
Fluoride	0.95 ± 0.59^Ab^	3.43 ± 1.00^Aa^	3.05 ± 1.22^Aa^
CPP-ACP	0.94 ± 0.62^Ab^	3.09 ± 1.57^Aa^	3.10 ± 0.88^Aa^
Nd:YAG laser + fluoride	1.13 ± 0.65^Ab^	2.72 ± 1.51^Aa^	2.79 ± 1.17^Aa^

Different uppercase letters indicate statistically significant differences within columns.

Different lowercase letters indicate statistically significant differences within rows.

Error between groups = 0,55.

Error intragroups: = 0,42.

*n* = 10, alfa 0,05.
